# Condom Negotiations among Female Sex Workers in the Philippines: Environmental Influences

**DOI:** 10.1371/journal.pone.0033282

**Published:** 2012-03-20

**Authors:** Lianne A. Urada, Donald E. Morisky, Nymia Pimentel-Simbulan, Jay G. Silverman, Steffanie A. Strathdee

**Affiliations:** 1 Division of Global Public Health, Department of Medicine, University of California San Diego, La Jolla, United States of America; 2 Department of Community Health Sciences, School of Public Health, University of California Los Angeles, Los Angeles, California, United States of America; 3 Department of Behavioral Sciences, College of Arts and Sciences, University of the Philippines Manila, Manila, Philippines; 4 Division of Global Public Health, Department of Medicine, University of California San Diego, La Jolla, California, United States of America; 5 Division of Global Public Health, Department of Medicine, University of California San Diego, La Jolla, California, United States of America; Vanderbilt University, United States of America

## Abstract

**Background:**

Social and structural influences of condom negotiation among female sex workers (FSWs) remain understudied. This study assesses environmental and individual factors associated with condom negotiation among FSWs at high risk for acquiring HIV in a large urban setting of Metro Manila, Philippines.

**Methods:**

Female bar/spa workers (N = 498), aged 18 and over, underwent interview-led surveys examining their sexual health practices in the context of their risk environments. Data were collected from April 2009-January 2010 from 54 venues. Multiple logistic regressions were conducted to assess socio-behavioral factors (e.g., age, education, length of time employed as an entertainer, and alcohol/drug use) and socio-structural factors (e.g., venue-level peer/manager support, condom rule/availability, and sex trafficking) associated with condom negotiation, adjusting for individuals nested within venues.

**Results:**

Of 142 FSWs who traded sex in the previous 6 months (included in the analysis), 24% did not typically negotiate condom use with venue patrons. Factors in the physical environment - trafficked/coerced into work (AOR = 12.92, 95% CI = 3.34–49.90), economic environment - sex without a condom to make more money (AOR = 1.52, 95% CI 1.01–2.30), policy environment - sex without a condom because none was available (AOR = 2.58, 95% CI = 1.49–4.48), and individual risk - substance use (AOR = 2.36, 95% CI = 1.28–4.35) were independently associated with FSWs' lack of condom negotiation with venue patrons.

**Conclusions:**

Factors in the physical, economic, and policy environments, over individual (excepting substance use) and social level factors, were significantly associated with these FSWs' condom negotiations in the Philippines. Drawing upon Rhodes' risk environment framework, these results highlight the need for policies that support safer sex negotiations among sex workers in the context of their risk environments. Interventions should reduce barriers to condom negotiation for FSWs trafficked/coerced into their work, substance using, and impacted by economic conditions and policies that do not support condom availability.

## Introduction

As the global sex industry expands, condom non-use among female sex workers (FSWs) remains an important target for HIV/STI prevention. Many studies focus only on individual-level constructs, which assumes the FSW has control over her environment and is the main person responsible for behavior change. However, other studies describe how FSWs' lack power in negotiating condoms with their clients [1]–[3] and that social and structural factors influence their behavior [4]–[6]. Morisky and his colleagues found that combined manager and peer interventions reduced STIs and risk behaviors among female bar entertainers in the southern Philippines [7]. However, less known are factors in the physical, social, policy, and economic environment that may influence condom negotiations specifically. This paper focuses on whether FSWs working as entertainers (term most commonly used to self-identify in this population) negotiate condom use with their clients (known as venue patrons), and examines the social and structural factors associated with condom negotiation.

Rhodes and his colleagues' [8], [9] developed a risk environment framework to address how harms in the physical, social, economic, and policy risk environments interact to influence the risk of HIV infection among substance users. This framework has been applied to FSWs [10]–[12] but not specifically to condom negotiation. Each physical, social, economic, and policy risk environment has a micro- and macro-level. Table 1 illustrates how this framework is defined and applied to the current study. For the physical risk environment, Rhodes assigned drug trafficking as a macro-physical risk and drug using location as a micro-physical factor. Likewise, sex trafficking in the current study is classified as macro-physical due to the organized nature of sex trafficking syndicates; recruiters usually target low income areas such as rural communities. Venue location or venue type is a micro-physical level variable. A study of injecting drug using FSWs, similar to this study, placed physical and sexual abuse as micro-physical risks [4]. Rhodes defines micro-social risks as social influences, such as peers, on substance use and HIV risk behaviors, while larger gender inequalities and stigmatization are classified as macro-social risks. Only micro-social level risks apply to the current study and are expanded to include peer and manager influences, interactions with sexual partners, and overall social support. Micro-economic risk level factors in Rhodes' and colleagues framework entails cost of living and health treatments and in the present study only micro-level economic risks are used to include income, cost of condoms sold at the venue, locations where the FSW obtains condoms, and frequency carrying a condom. For policy-level influences on HIV risk behavior, Rhodes includes availability of clean needles and syringes at the micro-level and public health policy governing harm reduction at the macro-level. In this study, only micro-policy factors apply and were modified to include condom availability and condom rule at the venue and frequency of receiving an HIV and STI test.

**Table 1:** Adaptation of Rhodes' Risk Environment Framework.

Environment	Rhodes' Definition	Measurement
Physical (macro)	Trafficking routes, population mobility	Have you ever been trafficked (tricked or forced) into a job as an entertainer?
Physical (micro)	Locations of risk activity	Workplace venue type: night club/bar, spa/sauna, karaoke center; Abuse: Have you ever experienced the following violence or abuse against you? (Physical or sexual)
Social (micro)	Peer group norms/local policing practices, community health and welfare service access/delivery	Peer support: Do you belong to an organization of workers? Has a co-worker ever discussed STI/HIV prevention with you? Has a co-worker (peer) at your venue ever tried to convince you to use a condom with a venue patron? Did you use a condom when having sex with venue patrons, due to your co-workers' advice or suggestion? If your STI symptoms do not go away, your peer/co-worker encourages you to get symptoms (STIs) treated? How frequently do you usually have contact with a peer at work?; Manager support: How supportive is your manager of your condom use with venue patrons? Has your manager ever talked to you about condom use? How frequently do you usually have contact with your manager at work?; Norbeck Social support Scale; Alcohol/substance use in the context of others: How often do you drink beer or alcohol with your venue patrons? How often are you drunk when you have sex? How often are your venue patrons drunk or high on drugs when they have sex with you?
Economic (micro)	Income, cost of living/health treatment/prevention materials	Average weekly income; Condom affordability: Where do you usually get condoms when you need them? How often do you carry a condom with you? Need/desire for income: How often do you have sex without a condom to make more money?
Policy (micro)	Availability of prevention materials, policies governing distribution/access	Condom availability: How often do you have sex without a condom because one was not available?; Venue policies: Does the venue you work in have a rule that all workers must use a condom when having sex with venue patrons? Are condoms available at your venue for the workers who work there? Testing: How often do you get an HIV or STI test?

### The Philippines Context

Prostitution is estimated as the fourth largest source of gross national product (GNP) for the Philippines [13]. Although illegal, many girls and women engage in sex work for lack of better options to support themselves and their families [14]. As many as 400,000 women and 100,000 children were forced or coerced into work annually within and across borders of the Philippines [15]. This paper addresses the role of trafficking, which has not yet been examined in relation to condom negotiation.

Although the Philippines is a low prevalence country (less than .1% infected of the adult population of 91 million), the HIV epidemic has steadily grown in recent years, with pockets of greater HIV concentration in certain areas and among high risk populations. For registered FSWs, a three-fold increase in HIV (68 to 230) occurred from 2007 through 2009 [16]. However, condom distribution remains under debate because of the Catholic Church's strong position against artificial contraception.

This study was situated is the largest urban setting in the Greater Metro Manila Area (GMMA), an area with 2.68 million people and nearly half of all reported HIV cases in the Philippines [17]. The Health Department mandates workers in night clubs, spa/saunas, and karaoke bars to register in local Social Hygiene Clinics (SHC) and to submit themselves for STI testing on a weekly or biweekly basis, and to attend an HIV 101 workshop upon start of employment. Some venues adopt a 100% condom use rule for workers to use condoms with venue patrons, and they fine or suspend workers if they do not comply. However, other venues fear authorities will perceive them as “unwholesome” if they have condoms at their venue.

We sought to determine the extent to which social and structural factors were associated with condom negotiation among female sex workers at high risk of acquiring HIV in this large urban setting of Metro Manila, the Philippines. We hypothesized that factors in the physical (more physical and sexual abuse and trafficking/force/coercion into work), social (less interactions with managers and co-workers, or more drinking with venue patrons), and policy (less condom availability and condom use rules) environments, and at the individual level (substance use and depression) were associated with not negotiating condom use with venue patrons. Understanding the factors associated with condom negotiation among FSWs in the Philippines may inform the design of HIV/STI prevention interventions for FSWs and have implications for policies at social and structural levels that support condom negotiation.

## Methods

### Ethics statement

Institutional Review Boards at the University of the Philippines Manila and the University of California at Los Angeles approved the study protocol. All participants involved in the study provided both verbal and written informed consent, documented on two separate consent forms. To protect the confidentiality of the participants due to the sensitive nature of the substance use and sex work questions and the absence of government certificates of confidentiality in the Philippines, the consent form with the participant signatures were only accessible for viewing by clinic staff for the purpose of matching names with clinic data. Only signatures of the witnessing interviewers were written on the other consent form and copies were given to the participant. The university ethics committees in both the U.S. and the Philippines approved this consent procedure.

### Study population

Women (n = 498), aged 18 and over, from 54 randomly sampled venues were interviewed in this cross-sectional study. The study identified venues in the community with workers registered at the two largest Social Hygiene Clinics in the city site within Metro Manila [12]. From a list of venues at each clinic, we categorized them according to three types (bar/night club/disco, karaoke bar, and spa/sauna), and proportionally sampled them according to size (number of workers) to obtain a closely matched sample between clinics. After stratifying by size and type, we randomly selected 4–6 venues of each type per clinic site, e.g. by selecting every third venue on the stratified list. All entertainers in each venue were interviewed (except for large venues where individuals were randomly sampled). Overall response rate was approximately 90%, with 10% of venue managers refusing to have their venue involved in the study.

From 498 entertainers, 173 reported trading sex in the previous six months, and 155 had complete data on the condom negotiation outcome variable (those with “not applicable,” “don't know,” or no answers were dropped). In the final regression model, 142 had complete data and were included in the analysis. Participants were from 21 venues (9 spa/saunas, 8 night clubs/bars, and 4 karaoke bars); 29% of the venues were from one clinic and 34% from the other clinic.

### Data collection

Non-government organization interviewers surveyed sex workers in private locations at the venue or SHC using structured questionnaires from April 2009-January 2010. Interviews, conducted in Filipino, lasted approximately 60 minutes. Participants received the equivalent of $3–5 USD in non-cash incentives of their choosing (i.e. stuffed animals, food).

### Measures

Interviewers surveyed participants on their sociodemographics, individual sexual risk and substance use behaviors, and experiences reflective of their physical, social, economic, and policy risk environments.

#### Sociodemographic

Questions included age, education, number of children, marital status, income, length of employment, length of time involved in the sex trade, number of sexual contacts in a typical week, number of STIs in the past 6 months, alcohol use, and substance use (frequency and type). A 24-item Center for Epidemiologic Studies Depression Scale (C-ESD) was used with a cutoff of 23 used in previous substance use research to indicate higher depressive symptoms [18], [19]; Cronbach's alpha was .79.

#### Physical environment

Following Rhodes' risk environment framework [8], [9], variables in the micro-physical environment included venue type (night club/bar, spa/sauna, and karaoke bar) and exposure to trauma (single items identifying if they “ever experienced physical or sexual abuse”). At the macro-physical level, participants were asked a single item, “Were you ever trafficked (forced or tricked) into your job as an entertainer?”

#### Social environment

Factors at the micro-social environment included peer and manager support using dichotomous measures previously validated in the southern Philippines [20], [21] (e.g., membership in an organization of workers, if an entertainer followed a co-worker's advice to use condoms, and if a manager supported condom use), and substance using behaviors taking place in the presence of others. Social support measured emotional, tangible, and functional support adapted from the Norbeck Social Support Scale (1995) [22], [23]. Cronbach's alpha for all items in the scale was .97. The validated scales were pretested (C-ESD and Norbeck Social Support) prior to use. The C-ESD was also previously validated with similar high risk groups in other countries [24], [25], while the Norbeck Social Support scale had not been widely used in FSW populations, but were tested in other high risk populations, e.g. HIV-positive substance users [26] and female-to-male transgender men [27].

#### Economic environment

Micro-economic items, previously used by Morisky and colleagues in the southern Philippines [20], [21], included weekly income, price of condoms sold at the venue, and where entertainers obtained condoms.

#### Policy environment

Micro-policy factors, also previously validated in the Philippines [20], [21] included condom rule at the venue, workplace provision of condoms, frequency of HIV and STI tests, and how often entertainers carried a condom.

#### Outcome variable

The outcome variable, condom negotiation, was measured by a single question “If a venue patron refuses to use a condom, what do you usually do?” for those trading sex in the past six months. Originally, this item was developed by using an open-ended question in Morisky and his colleagues' study of a southern Philippines population of FSWs. They found that intervention group participants identified more effective behaviors compared to a standard treatment group. The items were collapsed into a single closed ended question and tested in subsequent southern Philippines surveys [20], [21]. Individuals chose one of five responses: “Refuses to have sex with the venue patron (later coded as negotiates),” “Forces the venue patron to use a condom (negotiates),” “Explains the advantages of condoms (negotiates),” “Still has sex with the venue patron (does not negotiate),” and “only takes medication/treatment after sex (does not negotiate)”. The response choices were mutually exclusive since all respondents chose only one option for what they “usually” did when a patron refused to use condoms. If an FSW explained advantages of condoms, forced patrons to use condoms, or refused to have sex, then we assumed they actively reacted to the patron, whereas if they still had sex with the patron or only took medication/treatment after sex, we assumed they were not able to negotiate condom use.

### Analyses

Statistical analyses were conducted on FSWs who traded sex over the past six months, comparing those who usually negotiated condom use with those who did not, using t-tests for continuous and Pearson's Chi-square for non-continuous variables. Univariate and multivariate logistic regressions were performed to identify factors associated with condom negotiation, considering aforementioned factors at the micro and macro-level of the risk environment. Multi-level modeling controlled for individuals nested within venues. Models were developed using a manual procedure where all variables that attained a significance level <10% in univariate models were considered in multivariate analyses in order of most to least significance, and interactions were explored. To address potential collinearity among variables, the variables were put in the regression model one at a time, using a forward stepwise approach, and only retained if they remained statistically significant at the p<.05 level.

## Results

### Sociodemographics

As shown in Table 2, median age and duration in sex work were 23 and 12 months, respectively. They had a mean of 9 years of education, and 28% were married or had a live-in boyfriend. Self-reported prevalence of STIs during the past six months was 12%. They worked as an entertainer a median of 19 months, had 6 sexual contacts in a typical week, 70% used alcohol, and 17% used drugs. Three-quarters had CES-D depression scores of 23 or higher. In Table 3, 34% were ever physically abused, 44% sexually abused, and 13% were ever trafficked into jobs as entertainers. Their average weekly income was 6402 Pesos ($146 US).

**Table 2:** Sociodemographic, Biologic, Behavioral Characteristics associated with FSWs' Condom Negotiation with Patrons.

Baseline Characteristics	Total (n = 142)	Usually Negotiates (n = 107)	Does Not Usually Negotiate (n = 35)	T-Test/Chi-Square p-value	Odds Ratio (95%CI)
**Sociodemographics**					
Age (years)	23 (18–37)	23 (18–37)	23 (18–37)	0.964	1.00 (.90–1.10)
Education completed (years)	9 (1–15)	9 (1–15)	9 (3–12)	0.411	1.08 (.90–1.30)
Children	0.57 (0–4)	0.61 (0–3)	0.44 (0–4)	0.264	1.38 (0.78–2.45)
Marital status				0.952	0.97 (0.42–2.27)
Married or living with boyfriend	40 (28%)	30 (28%)	11 (30%)		
Living alone, separated, or widowed	102 (72%)	77 (72%)	26 (70%)		
**Biologic Factors**					
Had STI in the past 6 months^a^	11(12%)	9 (12%)	2 (7%)	0.376	2.05 (0.41–10.18)
**Lifetime Individual Risk Behaviors**					
Months worked as an entertainer	19 (1–156)	19 (1–156)	20 (1–74)	0.866	1.00 (0.98–1.02)
Months worked trading sex	12 (0–73)	11(0–48)	14 (1–73)	0.363	0.98 (0.95–1.02)
**Individual Current Risk Behaviors**					
Sexual contacts in a typical week	6 (0–36)	6 (0–36)	4 (0–10)	0.022	1.13 (1.01–1.27)*
Frequency of current substance use^b^ (sometimes = 1 to always = 5)	0.34 (0–3)	0.15 (0–2)	0.91 (0–3)	0.001	0.36 (0.22–0.59)*
Types of substance use^b^					
Shabu (crystal methamphetamine)	25 (18%)	13 (12%)	12 (32%)	0.003	0.27 (0.11–0.67)*
Other (marijuana, ecstasy)	18 (11%)	3 (11%)	5 (14%)	0.001	0.25 (0.11–0.58)*
Alcohol use frequency^c^	3.2 (1–5)	3.4 (1–5)	2.5 (1–5)	0.001	1.89 (1.37–2.60)*
CES-D Depression score	30 (8–55)			0.025	0.25 (0.07–0.90)*
< = 22	26 (25%)	23 (31%)	28 (90%)		
> = 23	78 (75%)	51 (69%)	59 (70%)		

* p<.05.

a self-reported.

b Yes-No responses on type/how often drugs used.

c never = 1, 1–2×/month = 2, 1×/week = 3, often not daily = 4, daily = 5; Note: Due to missing data, certain percentages may reflect denominators smaller than N given in the column head.

**Table 3:** Micro-Social Environmental Factors Associated with FSWs' Condom Negotiation with Venue Patrons.

Baseline Characteristics	Total (n = 142)	Usually Negotiates (n = 107)	Does Not Usually Negotiate (n = 35)	T-test/Chi-Square p-value	Odds Ratio (95% CI)
**Macro-Physical Risk Environment**					
Venue type (25 venues)				0.004	1.00 (0.68–1.48)
Night club/bar workers	50 (35%)	32 (30%)	18 (42%)		
Spa/sauna workers	71 (49%)	62 (58%)	9 (28%)		
Karaoke bar workers	21 (16%)	13 (12%)	8 (30%)		
Physically abused (ever)	40 (34%)	24 (29%)	16 (46%)	0.041	0.43 (0.19–0.98)*
Sexually abused (ever)	53 (44%)	35 (40%)	18 (57%)	0.161	0.56 (0.25–1.26)
Trafficked into job as entertainer (ever)	19 (13%)	6 (6%)	13 (35%)	0.001	0.10 (0.03–0.29)*
**Micro-Social Risk Environment**					
Peer/Co-worker supports for condom use					
Member of an organization of workers	15 (11%)	10 (9%)	5 (14%)	0.413	0.62 (0.20–1.95)
Peers discussed STIs	80 (56%)	60 (56%)	20 (54%)	0.913	0.96 (0.44–2.07)
Peer tried to convince to use condom	93 (65%)	70 (65%)	23 (62%)	0.975	0.98 (0.44–2.20)
Followed co-worker's condom advice	91 (64%)	81 (67%)	10 (57%)	0.565	1.26 (0.58–2.76)
Frequency of contact with co-workers	3.1(0–4)	2.9(0–4)	3.6 (0–4)	0.020	0.70 (0.50–0.97)*
Peer/co-worker encouraged STI treatment	74(47%)	57(66%)	17 (20%)	0.082	0.89(0.78–1.02)**
Manager supported condom use	62 (44%)	50 (47%)	12 (32%)	0.200	1.68 (0.76–3.72)
Manager discussed condom use with worker	42 (39%)	28 (36%)	14 (45%)	0.332	0.65 (0.28–1.53)
Frequency of contact with manager/owner	2.6 (0–4)	2.49 (0–4)	2.9 (0–4)	0.122	0.86 (0.67–1.11)
Overall social support^a^	76 (0–180)	71 (0–160)	92 (0–180)	0.024	0.99 (0.98–0.99)*
Frequency intoxicated while having sex^b^	1.67 (1–5)	1.51(1–4)	2.50 (1–5)	0.001	0.34 (0.21–0.54)*
Frequency drinks with venue patrons^b^	2.04 (1–5)	1.76 (1–5)	3.40 (1–5)	0.001	0.51 (0.39–0.67)*
Patron under influence of drugs while having sex^b^	1.87 (1–5)	2.02(1–5)	2.31 (1–5)	0.236	0.84 (0.63–1.12)

p<.05.

** p<.10.

a Sex workers had lower emotional, tangible social support (Norbeck scale) compared to a general sample.

b (never = 1, always = 5);Due to missing data, certain percentages may reflect denominators smaller than N in column head.

### Comparisons between FSWs who usually negotiated condom use vs. those who did not

#### Sociodemographics

For the 142 women included in the analyses, comparisons between those who usually negotiated condom use (76%) and those who did not (24%) suggested they were similar with respect to age, education, number of children, marital status, duration working as an entertainer and having paid sex, and number of STIs in the past six months. However, for individual risk behaviors, FSWs who did not usually negotiate condom use drank alcohol (p = .001) and used illegal substances more often (p = .001), had more depressive symptoms (p = .025), and more sexual contacts in a typical week (p = .022) than those who usually negotiated. Also, those who usually negotiated worried significantly more about getting HIV and had more perceived knowledge of STIs.

### Physical risk environment

#### Micro-physical

In Table 3, those who usually negotiated condom use vs. those who did not were not significantly different in their lifetime exposure to sexual abuse, but did differ with lifetime exposure to physical abuse (p = .041). The groups also differed according to type of venue; FSWs in night clubs/bars and karaoke bars negotiated condom use significantly less than those in spa/saunas (p = .004).

#### Macro-physical

Women who were trafficked into a job as an entertainer were less likely to negotiate condom use (p = .001).

### Social risk environment

#### Micro-social

The groups did not differ significantly on manager and peer support, social support, or whether venue patrons were high while having sex (Table 3). However, those who usually negotiated condom use differed from those who did not by having more contact with peers (p = .020), less total social support (p = .024), being less frequently intoxicated while having sex (p = .001), having less frequent drinks with venue patrons (p = .001), and being less certain if they had sex with an injecting drug user (p = .009).

### Economic risk environment

#### Micro-economic

Women who usually negotiated condom use did not differ significantly with respect to condom prices at their venue or income (Table 4). However, those who had sex without a condom to make more money were less likely to negotiate (p = .001) as well as those whose primary source for getting condoms were from drugstores, venue patrons or a friend/relative. Those who carried condoms more consistently (p = .016) were more likely to negotiate condom use than those who did not carry condoms as consistently.

**Table 4:** Economic and Policy Environmental Factors Associated with FSWs' Condom Negotiation with Venue Patrons.

Baseline Characteristics	Total (n = 142)	Usually Negotiates (n = 107)	Does Not Usually Negotiate (n = 35)	T-test/Chi-Square p-value	Odds Ratio (95% CI)
**Micro-Economic Risk Environment**					
Average weekly income (Pesos)	6402(500–30000)	6636(500–20000)	5643(1500–30000)	0.265	1.00 (0.99–1.00)
Primary place to get condoms					
Drugstore	56 (63%)	37 (56%)	19 (79%)	0.023	0.27 (0.08–0.88)*
SHC clinic	48 (68%)	38 (70%)	10 (56%)	0.382	1.67 (0.54–5.14)
Workplace venue	60 (75%)	49 (78%)	11 (61%)	0.188	2.15 (0.69–6.70)
Venue patron	27 (47%)	15 (36%)	12 (71%)	0.007	0.19 (0.05–0.68)*
Friend/relatives	14 (24%)	7 (17%)	7 (39%)	0.046	0.29 (0.08–1.00)**
Always carries a condom	56 (50%)	46 (57%)	10 (31%)	0.016	1.33 (1.03–1.72)*
Has sex without condom to make more money	1.9 (1–5)	1.61 (1–5)	2.94 (1–5)	0.001	0.46 (0.34–0.64)*
**Micro-Policy Risk Environment**					
Has sex without a condom because one was not available	1.6 (1–5)	1.3 (1–5)	2.4 (1–5)	0.001	0.38 (0.25–0.59)*
Venue has condom rule	68 (48%)	54 (50%)	14 (38%)	0.285	1.53 (0.70–3.32)
Venue provides condoms for purchase (average: 33 pesos per condom)	78 (55%)	62 (58%)	16 (43%)	0.210	1.64 (0.76–3.53)
Frequency of HIV test^b^	2.3 (1–5)	2.4 (1–5)	2.1 (1–5)	0.352	1.17 (0.84–1.62)
Frequency of STI test^b^	3.4 (1–5)	3.7 (1–5)	2.6 (1–5)	0.002	1.52 (1.15–2.01)*

* p<.05.

** p<.10; Note: Due to missing data, certain percentages may reflect denominators smaller than N given in the column head.

### Policy risk environment

#### Micro-policy

Women who usually negotiated condom use and those who did not were similar in their frequency of taking an HIV test and venue condom policies (Table 4). Overall, 48% had condom use policies and 55% had condoms available in their workplace. However, those who usually negotiated condom use had more frequent STI tests (p = .002) than those who usually did not negotiate condom use. Those who had sex without a condom because one was not available were also less likely to negotiate condom use (p = .001).

### Factors Independently Associated with Condom Negotiation

In a final multiple regression model (Table 5), factors independently associated with FSWs' lack of condom negotiation with venue patrons were: factors in the physical environment - trafficked/coerced into work (AOR = 12.92, 95% CI = 3.34–49.90), economic environment - sex without a condom to make more money (AOR = 1.52, 95% CI 1.01–2.30), policy environment - sex without a condom because none was available (AOR = 2.58, 95% CI = 1.49–4.48), and individual risk - substance use (AOR = 2.36, 95% CI = 1.28–4.35).

**Table 5:** Factors Independently Associated with FSWs Not Negotiating Condom Use with Venue Patrons, Controlling for Individuals Nested within Venues (n = 142).

Variable	Adjusted OR	Odds Ratio 95% CI
**Physical Risk Environment**		
Ever trafficked into job as an entertainer	12.92	3.34–49.90*
**Economic Risk Environment**		
Worker has sex without a condom to make more money^a^	1.52	1.01–2.30*
**Policy Risk Environment**		
Worker had sex without condom because none was available^a^	2.58	1.49–4.48*
**Individual Risk**		
Frequency of substance use^a^	2.36	1.28–4.35*

* p<.05.

a for those trading sex in past 6 months. Frequency of drug use is a crude measure. Missing n = 13: sex without condoms to make more money (n = 12), and whether they had sex without a condom because one was not available (n = 3).

## Discussion

This study of condom negotiation among FSWs working in a large urban setting of Metro Manila, Philippines, found that 24% did not usually engage in condom negotiation with their male clients. This finding is similar to other studies; 25% of FSWs in Vancouver, Canada reported being pressured by a client into not using condoms for sexual intercourse [28]. Similarly, studies of FSWs in Vietnam found that only half said clients consistently complied with their requests to use condoms [29] and successful condom use negotiation had a protective effect [30]. In the present study, trafficking (force/coercion into jobs as entertainers), the unavailability of condoms, and the need to make more money without a condom were significant structural factors related to not negotiating condom use. Substance use was also a factor contributing to not negotiating condom use among the FSWs. These findings highlight the need for macro- and micro-policy level changes, such as a stronger enforcement of the Anti-Trafficking in Persons Act of 2003 [31], increasing availability of condoms in venues and in the community, considering the impact of economics on an FSW's ability to negotiate condom use, and addressing substance use among the FSWs.

At the physical environment level, FSWs who were trafficked into their work were twelve times more likely to not negotiate condom use with their venue patrons than those who were not coerced/forced into their work. Except for a few studies [32], [33], the relationship between trafficking and condom use negotiation has not been studied. Women may not feel empowered to negotiate safer sex because they experienced less control over their work environments [1], [28]. The UN defines trafficking as those forced or coerced into sex, including minors under 18 years old engaged in the sex trade [34]. Only 3 women in this study revealed being underage when they first traded sex, but others identified being tricked or coerced into sex work. Trafficking legislation is critical at international and national levels, and more importantly, local government adherence to these laws, while being sensitive to the complexities of FSWs in the entertainment industry [35]. Philippine laws support anti-trafficking prosecution, but the Philippines remains on the U.S.' Tier-2 Watch List because of the continued trafficking syndicates operating in the country [36]. Our findings imply a need for stronger enforcement of the law and primary prevention of trafficking.

At the policy level, having sex without a condom because none was available was associated with not negotiating condoms. Other studies have found that having access to condoms was associated with safer sex practices among women who worked in bars in Tijuana, Mexico [37] and decreased unprotected sex among bathhouse patrons in Taiwan [38]. Studies underscore the importance of a supportive social environment for FSWs in China and India, such as a venue's condom availability, managerial support of condom availability, and clinic visitations [39], [40]. We therefore recommend policies requiring sex work venues in the Philippines to make condoms available to their workers. Five northern cities in the Philippines previously piloted 100% condom use policies [41], but such policies were not uniformly adopted in sex work venues. Venue managers need clarification that they will not be prosecuted for promoting 100% condom use policies. Likewise, efforts must be exerted to engage the Catholic Church to be more realistic and flexible in its stance on the use of artificial contraceptives, including condoms, similar to actions taken by civil society groups in the campaign to have the Reproductive Health Bill passed into Philippine law [42]. The church's interference of HIV prevention efforts continues to be a center of debate in the Philippines and other settings, despite the Vatican Pope's more recent approval of condom use to fight AIDS [43].

The fact that the women had sex without a condom to make more money illustrates continued economic environment risks that impact condom negotiation. Likewise, sex workers in other countries like India and Mexico feared losing income if they negotiated [1], [4]. Making condoms free, not just available, may help in situations where condoms are already difficult to negotiate due to economic pressures. DKT International, a non-government organization has sold condoms in the country at 5 Pesos through its social marketing campaigns, compared to the average cost sold at the venue of 33 Pesos [44]. Also, developing options such as involving FSWs in community participatory research methods and grant-writing may lead to additional income. For example, the Peer Educators Movement for Empowerment of Pasay, Manila, Caloocan and Quezon City, involved in facilitating this research, may serve as a model. Funded by UNICEF, the Philippine Rural Reconstruction Movement trained the peer educators, a group of at-risk youth composed of FSWs, gang members, in-and-out-of-school youth, and males having sex with males. Besides the cost of condoms, condom availability, and income differences, other factors may influence the women's decisions to have sex without a condom to make more money and needs further study.

FSW's substance use in this study also negatively impacted their condom negotiations with venue patrons, more than other individual factors (i.e. alcohol use, depression, age). In particular, methamphetamine use (“shabu”) was high (18%) among those using substances. Other studies have documented the association between substance use and condom negotiation among FSWs [45], [46]. In the Philippines, injecting drug use is increasing and methamphetamine use has been the drug of choice for 90% of substance users. A 2008 World Drug Report of the United Nations Office on Drugs and Crime (UNODC) said the Philippines, at 6%, had the highest estimated annual methamphetamine prevalence rate worldwide [47]. This has implications for interventions around crystal methamphetamine use, the drug of choice used most among FSWs. More research is critical to determine how to intervene with substance using FSWs.

Other hypotheses involving certain micro-social variables (e.g., client-level variables and peer support) were not supported by this study. For example, venue patrons under the influence of drugs while having sex, frequency of intoxication while having sex with venue patrons, drinking with venue patrons, having sex with an injecting drug user, and peer support were not significantly associated with condom negotiation in the final model. Findings indicate that physical, economic, and policy environmental factors may influence condom negotiation more than peer interaction, manager support, and sexual partner characteristics. However, in the binary and multivariate analyses, most social and structural factors were not significantly associated with condom negotiation. For example, factors such as peer support and manager contact were associated with consistent condom use in another analysis [12], but these factors did not emerge for condom negotiation. Training peers and managers on condom negotiation skill-building may be necessary, as in Hong Kong where manager pressure had a negative effect on FSWs' condom self-efficacy [6]. However, FSWs at highest risk should be targeted: those ever trafficked, substance using, and those who work in environments where condoms are not available or where there is greater pressure to make money without condoms, as these barriers appear to have a greater association with not negotiating condom use than other factors, e.g. peer, manager, and sexual partner support.

This study is limited by its cross-sectional design which cannot infer causal relationships. Measures were entirely based on self-report, posing another potential limitation. Sex workers may have under-reported their venue policy's influence on condom use due to the laws against prostitution. The measures of substance use were relatively non-specific and may have led to under-reporting. Further qualitative inquiry might capture other nuances of condom negotiation beyond what the FSW might do if a venue patron refused to use condoms. For example, in Singapore, FSWs initiated condom use, but highly educated clients were more difficult to persuade to use condoms than lower educated clients [2]. Familiarity with clients also posed barriers to condom negotiation in another FSW study conducted in China [48]. The associations between venue patron types and FSW condom negotiations may warrant further attention.

These findings have salient implications for structural interventions that are needed to interrupt HIV transmission behaviors among FSWs [4], [49], [50], [51], [52]. Policy constraints, such as slow implementation of anti-sex trafficking legislation and continued debate about making contraceptives available in the Philippines places FSWs at high risk for their not negotiating condom use. Recommendations may receive opposition by non-supportive leaders in government and church leaders. Indeed, reorienting non-supportive legislators' and the Catholic Church's stance on condom use in the context of the HIV/AIDS epidemic and over-population problem in the Philippines is a major structural intervention worth pursuing. Furthermore, interventions are needed to address the economic realities of women in the sex trade along with the urgent need for more substance use interventions and research in the Philippines. Findings suggest that structural interventions at the policy and venue level should be integrated into interventions that build safer sex negotiation skills among sex workers.
